# Gaussian Process Regression for Single-Channel Sound Source Localization System Based on Homomorphic Deconvolution

**DOI:** 10.3390/s23020769

**Published:** 2023-01-09

**Authors:** Keonwook Kim, Yujin Hong

**Affiliations:** Division of Electronics & Electrical Engineering, Dongguk University-Seoul, Seoul 04620, Republic of Korea

**Keywords:** Gaussian process regression, sound source localization, single channel, time of flight, angle of arrival, homomorphic deconvolution, cepstrum, machine learning, Yule–Walker, Prony, Steiglitz–McBride, similarity matrix

## Abstract

To extract the phase information from multiple receivers, the conventional sound source localization system involves substantial complexity in software and hardware. Along with the algorithm complexity, the dedicated communication channel and individual analog-to-digital conversions prevent an increase in the system’s capability due to feasibility. The previous study suggested and verified the single-channel sound source localization system, which aggregates the receivers on the single analog network for the single digital converter. This paper proposes the improved algorithm for the single-channel sound source localization system based on the Gaussian process regression with the novel feature extraction method. The proposed system consists of three computational stages: homomorphic deconvolution, feature extraction, and Gaussian process regression in cascade. The individual stages represent time delay extraction, data arrangement, and machine prediction, respectively. The optimal receiver configuration for the three-receiver structure is derived from the novel similarity matrix analysis based on the time delay pattern diversity. The simulations and experiments present precise predictions with proper model order and ensemble average length. The nonparametric method, with the rational quadratic kernel, shows consistent performance on trained angles. The Steiglitz–McBride model with the exponential kernel delivers the best predictions for trained and untrained angles with low bias and low variance in statistics.

## 1. Introduction

The delivered signal over the space contains the spatial information due to the limited propagation speed. The walls and/or obstacles in the field may provide multipath patterns on the received signal. In the isotropic and open environment, the multiple receivers create numerous arrivals on the received signal. The propagated signal can be analyzed by the sound source localization (SSL) system to estimate the angle of arrival (AoA) for the signal source. The first engineering approaches for the SSL system utilized the unidirectional sound collection structure with steering for localizing. To avoid the physical movement, the extensive processing over the multichannel signals is required to understand the spatial information. The mathematical modeling of the propagation and arrival of the sound is important for processing in terms of sound directions.

The conventional method for SSL is beamforming [1,2], which uses the phase information from the multiple receivers for the spatial filtering. The accuracy of the beamforming localization is proportional to the receiver numbers along with the processing power; therefore, the significant computing power as well as data rate are involved in the precise sound localization. Certain natural creatures, including humans, can accurately localize sound sources in three-dimensional (3D) space by using the binaural correlation and structure profile [3]. The reflections and diffractions on the receiver structure provide additional features to derive the AoA for the sound sources. The human hearing for localization has been imitated by the SSL system based on the monaural [4] and binaural [5] configurations. With reduced receivers and computation, the researchers are continuously conducting investigations to understand the complex propagation of the physical structure for precise and feasible SSL systems [6,7,8,9,10,11].

The recently developed autonomous driving systems comprehend their surroundings through camera vision and active sensors, which deliver pinpoint accuracy for localization. The general driving conditions frequently present the situations beyond the immediate hazards and warnings above the direct detection and estimation. The acoustic information can be employed for elevating the safety of the autonomous system further due to the acoustic signal properties [12]. The sound source can be identified and localized by the SSL system based on the reflected and diffracted propagation for the extended ranges. Therefore, upon the detection of the direct or indirect endangerment, the system may activate the pre-emptive safety procedures to reduce the possibility of the imminent accidents. Obviously, the sound is complementary information to the vision for navigating with mobile and immobile objects. We, as humans, completely perceive the importance of hearing for everyday navigation and communication. The loss of sound localization capability makes a person unable to understand the environment via visual information.

The SSL system demands the multiple arrival information in the form of a time delay in the received signal. The beamforming algorithms explicitly obtain the time delays from multiple and independent receiver channels [1,2]. Therefore, the dedicated and synchronized communication channels should be designed to avoid temporal contamination. The biomimetic-inspired SSL systems, such as monaural and binaural methods, figure out the AoA based on the structure around the receiver to implement the subtle time delays in the received signal [4,5]. The dedicated communication as well as additional structure prevent the realization of the SSL system in the mobile objects due to the complexity and safety. Additionally, the receivers installed over the 3D surface create further complexity to derive the sound propagation model, which is the fundamental component of the conventional SSL system. For the autonomous mobile system, the novel SSL method should be developed to achieve optimal, scalable, and feasible performance.

Continuously, numerous novel SSL systems have widely utilized machine/deep learning algorithms to improve their performance and adaptation in various deployment environments. SongGong et al. [13] present the frequency-invariant circular harmonic features for the convolutional neural network to obtain an accurate AoA estimation. Nguyen et al. [14] suggest the feature based on the multichannel log-spectrograms and normalized principal eigenvector of the spatial covariance matrix for the convolutional recurrent neural network to estimate and detect the AoA and event, simultaneously. In the spherical microphone array, the spherical map representation with a dual-branched convolutional autoencoder is proposed for the multiple sound source localization [15]. Tan-Hsu, Yu-Tang, Yang-Lang, and Mohammad provide the convolutional neural network with a regression model to estimate the sound source angle and distance based on the interaural phase difference [16]. To improve SSL performance in noisy and reverberant environments, the steering vector phase difference enhancement is realized by the deep neural network [17]. The SSL system invariant to the configuration was developed by Chun et al. based on the azimuth-frequency representation and convolutional neural networks [18].

The machine/deep learning-based SSL system extends the application to a specific area. The flying drone localizes the sound source from ambient noise and frequent movement [19]. The low-power device with a microphone array derives the AoA for the sound source in the application of various human interaction [20]. The complex environment from the indoor condition is challenged for the SSL system by Machhamer [21] and Zhang [22]. The sound source-specific classification and localization are accomplished for the gunshot [23], acoustic alarm [24], and multispeaker [25]. The underwater SSL system is implemented by comprehending the complicated sound propagation based on the convolutional neural networks [26], artificial neural networks [27], and deep neural networks [28]. Grumiaux, Kitić, Girin, and Guérin organized the survey on deep learning methods for single and multiple SSL in indoor/domestic environments with an exhaustive topography [29].

This paper proposes a single analog-to-digital converter (ADC) channel SSL system with multiple receivers. The multiple arrivals in the received signal are superimposed into one data flow for single-channel processing. The receiver locations are configured to maximize the pattern diversity in a time-delay distribution. The deconvolution procedure from the single channel data extracts the time delays, which are produced by the receiver configuration for direction-wise time delays. The time delays can be represented by the nonparametric and parametric features of the machine learning algorithm. The supervised machine learning for the regression predicts the AoA based on feature learning. The benefits of the proposed single-channel sound source localization (SCSSL) system can be described as scalability and feasibility. The increased number of receivers in the SCSSL system contributes to the enhanced prediction accuracy without expanding the data rate or computation requirements. The proposed SCSSL system demonstrates the constant computational complexity in terms of receiver number due to the unique data and computation flow. Additionally, the SCSSL system does not require an explicit mathematical model for the temporal arrival information due to supervised machine learning. In all practicality, the receivers can be placed and attached at any place as long as the receivers are located within the common wire extension range. Note that all receivers are connected based on the bus topology using a simple analog network with the analog mixer.

**Figure 1:**
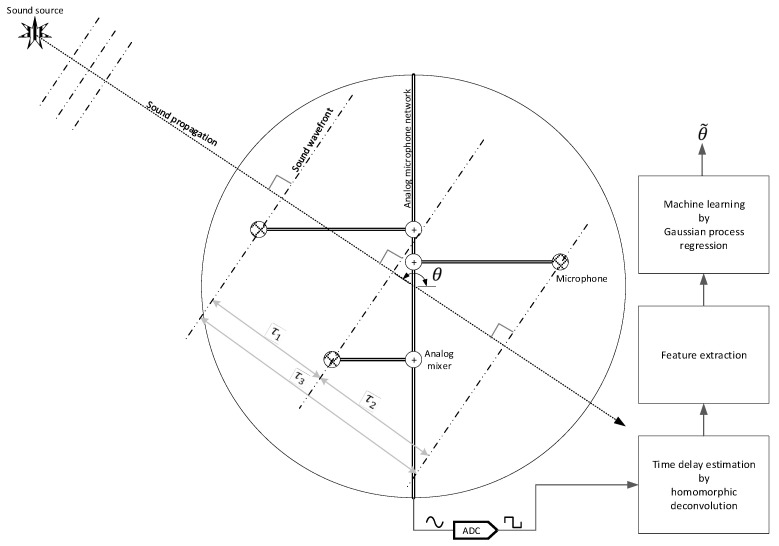
Functional diagram of the overall SCSSL system. The big circle indicates that the object installed three microphones with an analog microphone network. θ is the real arrival angle for the sound and $\tilde{\theta }$ is the estimated angle.

The SCSSL system consists of three computational stages: homomorphic deconvolution (HD), feature extraction, and machine learning stage, as shown in Figure 1. The sound source emits the acoustic waves in spherical shape, and the wavefronts arrive at the receiver module in plane format with far field provision. According to the AoA θ, the inter-receiver time delays $\tau_{1},\;\tau_{2},\;\mathrm{and}\;\tau_{3}$ are determined and fused as independent arrivals on the analog microphone network. The single ADC transforms the mixed analog signal into digital format for further processing. The HD separates the time delays (or impulse responses) of the receiver module from the single-channel received signal. The previous paper confirmed the performance of time of flight (ToF) estimation by utilizing the nonparametric and parametric HD algorithms [30]. The nonparametric HD presents the raw distribution of time delays, and parametric HDs show the parametrized rational function of the time delays. Therefore, the feature extraction stage isolates the representative information from the given HD outcome by selecting the proper domain. The Gaussian process regression (GPR) in the machine learning stage performs the stochastic process on the supervised learning to define the likelihood distribution over functions. Proper parameter estimation and Bayesian function modeling can predict the accurate arrival angle based on the given features. The increased number of receivers is expected to produce improved accuracy in the prediction; however, this paper uses the minimum number of receivers as three in the horizontal space.

This paper justifies the author’s continuous research activities on the SSL system for decades. The novel underwater SSL systems based on the beamforming had been presented for rapid and scalable systems [31,32,33,34,35,36]. The sparsely distributed wireless sensor network localized the targets by capturing the acoustic energy from the moving vehicle [37]. The unique binaural [38] and monaural [39,40,41] SSL systems were proposed for feasible systems over the airborne sound propagation. The human aggressively exploits the binaural and monaural methods for better localization by using the magnitude, phase, and frequency variations over the two ears as well as the pinna/head structure [3]. The binaural and monaural SSL systems are possible because of the extensive use of the structure to overcome the receiver number limitation. In general, the conventional sensing modules are required to be installed on the target system without modifying the overall structure and configuration. Additionally, the computation and communication complexity should be maintained at a low level for system feasibility. The single-channel multiple-receiver SSL was suggested in the previous papers [30,42] to challenge the binaural/monaural method without the external structure and to include the beamforming performance with reduced computation/communication burden. The paper established the promising performance with a simple machine learning algorithm, linear regression. This paper extends the single-channel, multiple-receiver SSL method with an advanced machine learning algorithm called GPR and novel feature extraction. Additionally, the simulation and experiment are diversified by including the further hold-out dataset for extensive angles. Therefore, this paper investigates the prediction performance along with the complex inference of the angles not shown in the learning process. Note that the identical anechoic chamber [43] has been operated for the acoustic experiments and evaluations to maintain consistency.

This paper is organized as follows: Section 2 introduces HD and explains how the time delays are extracted and represented. Section 3 presents the feature extraction methods for nonparametric/parametric HDs and the GPR for machine learning. Section 4 presents the computer simulations for the optimal receiver position and prominent localization parameters. The similarity matrix is employed for evaluating the various receiver positions with reduced computation. The simulation also demonstrates the performance of localization for the numerous parameters and models. The prediction performances on the training and hold-out datasets are illustrated and analyzed for extensive directions. Section 5 describes the performance of the actual dataset produced by the anechoic chamber experiments. The best parameters and model benefits are observed and arranged for the conclusion.

## 2. Nonparametric and Parametric Homomorphic Deconvolution

This section is presented by summarizing the previous papers [30,42] to provide access to the reader while maintaining concept integrity. For further information, the readers are suggested to follow the article for finding detailed explanation about the nonparametric and parameter HD. The homomorphic filtering method is a comprehensive method for signal processing, comprising a nonlinear transformation to a counterpart domain in which linear filters are performed, followed by an inverse transformation back to the original domain [44,45,46]. The HD is one application of homomorphic filtering to separate the convoluted signals by using the logarithm and exponential operations. The forward homomorphic system based on the logarithm converts the multiplicative functions into an additive format. The simple window operation performs the separation based on a signal property. The backward homomorphic system with exponentials reverts to the original time domain. The HD uses two homomorphic systems in a cascade to derive the propagation function, which indicates ToFs between the receivers.

**Figure 2:**
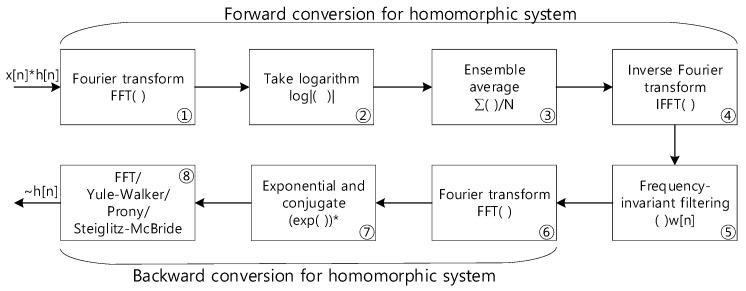
Computational procedure for the parametric and nonparametric homomorphic deconvolution. The * in stage ⑦ indicates the conjugate operation.

The nonparametric HD produces the conventional raw distribution of time delay information based on multiple discrete Fourier transforms (DFT) or fast Fourier transforms (FFT). The propagation function model realizes the parametric ToF estimation in the last HD stage by employing Yule–Walker [47,48], Prony [48,49], and Steiglitz–McBride [48,50]. Note that the parametric method computes model coefficients and the nonparametric technique delivers the numerical distribution. Figure 2 illustrates the complete computational procedure for the nonparametric and parametric HD algorithms. The ensemble average in stage ③ and conjugate in stage ⑦ are inserted to conduct the signal-to-noise ratio (SNR) improvement and parametric modeling, respectively.

In Figure 2, the system receives the single channel signal, which contains the impulse response (or propagation function) of the receiver configuration based on the convolution sum. Note that the star operation in $x\left[n\right]*h\left[n\right]$ in Figure 2 indicates a convolution sum operation. The FFT (stage ①) and inverse FFT (stage ④) pair with the absolute logarithm (stage ②), which corresponds to the real cepstrum of the forward conversion of the homomorphic system. The previous paper [42] showed that the ensemble average (stage ③) after the logarithm (stage ②) denotes the inverse proportionality in noise variance. Therefore, the longer ensemble average length induces higher SNR due to the lower noise power. The window function $w\left[n\right]$ in frequency invariant filtering (stage ⑤) actually executes the separation of impulse response $h\left[n\right]$. The $w\left[n\right]$ is allowed to pass beyond the specified time sample, which controls the minimum time delay in $h\left[n\right]$. The other FFT (stages ⑥ and ⑧) pairs with exponential functions (stage ⑦) reconstitutes and estimates the nonparametric propagation impulse response $\tilde{h}\left[n\right]$. The FFT in the last stage essentially performs the inverse FFT because the conjugate to the FFT provides the conjugated inverse FFT outcome according to the DFT/FFT property.

The choice of the last computation stage in Figure 2 is divided between the nonparametric and parametric HD. The Yule–Walker, Prony, and Steiglitz–McBride method are devised to provide the parametric HD. The following equations show the conventional regressive signal models in the time and *z* domains.

(1) $$y\left[n\right]+a_{1}y\left[n-1\right]+\ldots +a_{N}y\left[n-N\right]=b_{0}x\left[n\right]+b_{1}x\left[n-1\right]+\ldots +b_{M}x\left[n-M\right]$$

(2) $$H\left(z\right)=\frac{b_{0}+b_{1}z^{-1}+\ldots +b_{M}z^{-M}}{1+a_{1}z^{-1}+\ldots +a_{N}z^{-N}}$$

The model parameters below are estimated by the Yule–Walker algorithm to describe the propagation function $h\left[n\right]$ of the given time signal. The Yule–Walker algorithm is classified as the autoregressive (AR) model; hence, the algorithm derives the coefficients of the denominator in Equation (2).

$$\left\{{\tilde{a}}_{1},{\tilde{a}}_{2}\ldots ,\;{\tilde{a}}_{N}\right\}\in \mathbb{C}\;\mathrm{and}\;b_{0}={\tilde{\sigma }}^{2},\;b_{1}=b_{2}=\ldots =b_{M}=0$$

The model coefficients below are computed by the Prony and Steiglitz–McBride algorithms to represent $h\left[n\right]$. The Prony and Steiglitz–McBride algorithms belong to the autoregressive moving average (ARMA) model; therefore, the algorithms are required to compute the numerator and denominator coefficients in Equation (2).

$$\left\{{\tilde{a}}_{1},\ldots ,\;{\tilde{a}}_{N},\;{\tilde{b}}_{0},\;\ldots ,\;{\tilde{b}}_{M}\right\}\in \mathbb{C}$$

Note that the complex number input for the Yule–Walker, Prony, and Steiglitz–McBride algorithm provide the complex number coefficients which disengage the pole/zero location constrains. Without the constraints, further estimation accuracy is obtained from the numerical expansion [30].

(3) $${\left|\tilde{h}\left[n\right]\right|}^{2}={\left|{\left.H\left(z\right)\right|}_{z=e^{j2\pi n/L}}\right|}^{2}$$

In Equation (3), the estimated time delays are properly indicated by the derived coefficients from the parametric methods. The $H\left(z\right)$ is the rational function shown in Equation (2), and *L* is the consistent FFT length in the HD algorithm. Observe that the samples *n* corresponding to the maximum propagation function $\tilde{h}\left[n\right]$ specify the time delays.

**Figure 3:**
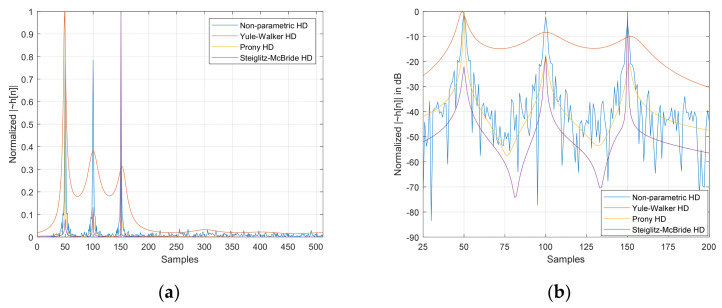
Estimated $\left|\tilde{h}\left[n\right]\right|$ distribution (normalized), with 200 ensemble average lengths for 50, 100, and 150 sample delays. The order for the parametric HDs is 9 and the window length for all HDs is 25 samples. (**a**) Magnitude distribution; (**b**) decibel distribution. $\tilde{h}\left[n\right]$ is represented by $\sim h\left[n\right]$.

Figure 3 illustrates the normalized $\left|\tilde{h}\left[n\right]\right|$ for 50, 100, and 150 samples in time delay for the nonparametric and parametric HD algorithms. The window length for all HDs is 25 samples, with a 200 ensemble average length. The order for the parametric HDs is 9 in constant. The data is generated from the recorded audio signal from the anechoic chamber [43] with postprocessing (convolution sum) for dedicated time delays. The HDs demonstrate the statistical performance based on the SNR, ensemble average length, and order (only for parametric HDs). The nonparametric HD and Steiglitz–McBride methods provide consistent estimation with low variance and bias in general. The Yule–Walker and Prony methods are prone to the performance degradation in low SNR and short ensemble average length overall. Further statistical performances were analyzed for time delay estimation in the previous paper [30] based on the various conditions.

## 3. Methodology

The previous section introduced the methods to derive the time delay information, which is induced by the receiver structure. The process is known as the deconvolution for reversing the convolution between the original sound and produced delays. Based on the delay distribution, the AoA is estimated by the machine learning algorithm, the GPR with proper information format. The GPR predicts the AoA based on the supervised learning process, which computes the optimal function from the statistical procedure. The nonparametric and parametric HD algorithms produce distinctive numerical distribution for the time delay; therefore, the appropriate feature extraction is necessary for the GPR. The following subsections demonstrate the feature extraction and GPR methods for accurate predictions.

### 3.1. Feature Extraction

Machine learning algorithms classified as supervised require labeled examples with feature input. The regression problem predicts a real-valued label from the feature learning process for the given unlabeled feature input. The GPR is one of the supervised and regression algorithms; therefore, a representative feature dataset as well as accurate labels are necessary for the learning process. The features and labels are arranged as follows:

(4) $${\left\{\left(x_{i},y_{i}\right)\right\}}_{i=1}^{L}\;\mathrm{where}\;x_{i}\in {\mathbb{R}}^{M\times 1},\;y_{i}\in \mathbb{R}$$

The lower case presents the scalar and the bold lower case denotes the vector for the *L* dataset in the above equation. The subscript indicates the *i*-th dataset. The label $y_{i}$ is produced by the feature vector $x_{i}$. The individual vector ***x****_i_* is organized as follows:

(5) $$x_{i}={\left[x_{i}^{\left(1\right)}\;x_{i}^{\left(2\right)}\ldots \;x_{i}^{\left(M-1\right)}\;x_{i}^{\left(M\right)}\right]}^{T}$$

The *n*-th feature in the vector is described by the superscript number *n* in parenthesis in the above equation. Note that there are *M* features in the vector. The choice of $x_{i}$ is distinctive for the nonparametric and parametric HD algorithms in terms of domain as well as length.

The nonparametric HD utilizes the *direct method* for feature extraction. The raw distribution of nonparametric HD output immediately illustrates the time delay information with likelihood. The higher value in the sample location shows the stronger possibility of time delay in the sample location. The range of the distribution follows the time delay information from the given receiver configuration. The lower bound *W* is specified by the window function $w\left[n\right]$ in Figure 2 stage ⑤ since the window function prunes the time delay below the cutoff *W* to separate the ToF from the received signal.

(6) $$x_{i}^{\left(l\right)}=\tilde{h}\left[l+W\right]\;1\leq l\leq n_{max}-W$$

Equation (6) shows the feature extraction from the $\tilde{h}\left[n\right]$ nonparametric HD output. The $n_{max}$ is the maximum time delay possibly derived from the receiver configuration. The time locations from the *W* + 1 to $n_{max}$ of the nonparametric HD output are organized for the feature $x_{i}^{\left(l\right)}$ sequentially. Note that the $\tilde{h}\left[n\right]$ nonparametric HD output is normalized for consistency.

The parametric HDs employ the parameter-based extraction method known as *pole angle method* for feature extraction. The parametric HDs can produce the same raw distribution as the nonparametric HD output by using Equation (3); however, the benefits of the parametric HDs are lost with the extra computation. All parametric HDs establish the rational function as shown in Equation (2), and the poles of the function are the dominant components to create the peaky response in the time delay distribution. Especially, the phases of the poles correspond to the time delay in HD distribution [30]. The denominator of the rational function in Equation (2) is shown below, and the solution of the polynomial provides the poles of the given regressive model.

(7) $$1+{\tilde{a}}_{1}z^{-1}+{\tilde{a}}_{2}z^{-2}+\ldots +{\tilde{a}}_{N}z^{-N}=\left(1-\mu_{1}z^{-1}\right)\left(1-\mu_{2}z^{-1}\right)\ldots \left(1-\mu_{N}z^{-1}\right)$$

The polynomial coefficients ${\tilde{a}}_{i}$ in the above equation are estimated parameters by using the Yule–Walker, Prony, and Steiglitz–McBride methods with the given order *N*. The ${\tilde{a}}_{i}$s are complex numbers, and the solutions $\mu_{i}$ of the polynomial are also complex numbers. The phase of the individual pole $\mu_{i}$ is assigned to the feature vector, as shown below.

(8) $$x_{i}^{\left(l\right)}=∡\mu_{l}\;\mathrm{for}\;1\leq l\leq N-\pi <∡\mu_{l}\leq \pi$$

The ∡ operator computes the angle of the complex number $\mu_{i}$ by using the simple trigonometric calculation in radians.

The direct method is the simplest method to extract the feature from the HD output. The method simply extracts the portion of the output and cast it on the feature vector. The length of the feature vector from the direct method is likely to be extensive due to the maximum time delay collected by the receiver configuration. The pole angle method requires further computation to calculate the poles and phases. However, the length of the feature vector from the pole angle method is expected to be concise because of the parametric model order. Observe that the parameter order is considerably smaller than the HD length, and the model parameters represent the time delay based on the dedicated computation introduced in Section 2.

### 3.2. Gaussian Process Regression

The Gaussian process regression contains two methods: the Gaussian process and regression. By definition, a Gaussian process is a collection of random variables, any finite number of which have a joint Gaussian distribution. Therefore, the Gaussian process generalizes the Gaussian distribution to an uncountably infinite set of possible dimensions. The regression is a problem of predicting a real-valued output given a feature vector by establishing a model from a learning algorithm. The GPR assumes that the regression outputs are infinite and latent function variables $f\left(x_{i}\right)$ and the finite collection of the function variables obeys the multivariate Gaussian distribution. The Bayesian modeling of functions allows us to train the model and predict the target, especially in applications where data is limited.

The GPR is an extensive research area for the machine learning algorithm utilized for various purposes [51,52,53]. The diverse approaches have explained the GPR in numerous papers [54,55] and web documentation [56,57] in mathematical and technical terms, respectively. Note that the following equations and descriptions provide the GPR for the given application/structure in a concise and deliverable style. The function $f\left(x\right)$ below is specified by the Gaussian process with zero mean and input variance. Observe that the function variance is computed by the input variance [58,59].

(9) $$f\left(x\right)\sim gp\left(0,k\left(x,x\right)\right)\;x\in {\mathbb{R}}^{M\times 1}$$

The explicit basis function is a method to denote a nonzero mean over the function. The equation below represents the zero-order mean basis function 1 and coefficient β with the given mean and variance for the coefficient. The higher order mean function can be realized by the mean function matrix with vector β.

(10) $$g\left(x\right)=f\left(x\right)+\beta \;\beta \sim N\left(b,B\right)$$

The GPR output $g\left(x\right)$ in the equation below shows the Gaussian process with the new mean and variance for the linear combination in Equation (10).

(11) $$g\left(x\right)\sim gp\left(b,k\left(x,x\right)+B\right)$$

The scalar output of Equation (11) is extended to the vector output in the equation below. The bold font in the function name presents the vector output. The h corresponds to the basis function and one vector for the zero order.

(12) $$y=g\left(X\right)=f\left(X\right)+h\beta$$

The matrix/vector format and dimension are demonstrated in below equation. Note that the $x_{i}$s in below equation are the feature vectors specified at Equation (5).

(13) $$y=\left[\begin{array}{c} y_{1}\\ y_{2}\\ \vdots \\ y_{L} \end{array}\right]\;g=\left[\begin{array}{c} g\left(x_{1}\right)\\ g\left(x_{2}\right)\\ \vdots \\ g\left(x_{L}\right) \end{array}\right]\;f=\left[\begin{array}{c} f\left(x_{1}\right)\\ f\left(x_{2}\right)\\ \vdots \\ f\left(x_{L}\right) \end{array}\right]\;X=\left[\begin{array}{c} x_{1}^{T}\\ x_{2}^{T}\\ \vdots \\ x_{L}^{T} \end{array}\right]\;h=\left[\begin{array}{c} 1\\ 1\\ \vdots \\ 1 \end{array}\right]\in {\mathbb{R}}^{L\times 1}$$

The joint distribution between the observed y and predicted $y_{*}$ function values derives the conditional distribution with $\bar{g}\left(X_{*}\right)$ mean and $cov\left(g\left(X_{*}\right)\right)$ covariance, as shown below. $I_{L}$ is the identity matrix for *L* size.

(14) $$y_{*}=\bar{g}\left(X_{*}\right)=h_{*}\beta +K\left(X_{*},X\right){\left(K\left(X,X\right)+\sigma_{n}^{2}I_{L}\right)}^{-1}\left(y-h\beta \right)$$

(15) $$cov\left(g\left(X_{*}\right)\right)=K\left(X_{*},X_{*}\right)-K\left(X_{*},X\right){\left[K\left(X,X\right)+\sigma_{n}^{2}I_{L}\right]}^{-1}K\left(X,X_{*}\right)\;+{\left(h_{*}^{T}-h^{T}{\left(K\left(X,X\right)+\sigma_{n}^{2}I_{L}\right)}^{-1}K\left(X,X_{*}\right)\right)}^{T}\left(h^{T}{\left(K\left(X,X\right)+\sigma_{n}^{2}I_{L}\right)}^{-1}h\right)\;\;\left(h_{*}^{T}-h^{T}{\left(K\left(X,X\right)+\sigma_{n}^{2}I_{L}\right)}^{-1}K\left(X,X_{*}\right)\right)$$

The dimensions of the predicted output and basis function are shown in the equation below. The actual prediction of the GPR is the mean of the conditional distribution, $y_{*}$ equivalently $\bar{g}\left(X_{*}\right)$. $L_{*}$ is the length of the test dataset.

(16) $$y_{*}\in {\mathbb{R}}^{L_{*}\times 1}\;h_{*}\in {\mathbb{R}}^{L_{*}\times 1}$$

The computation of the prediction $y_{*}$ requires hyperparameters as covariance matrix, β, and $\sigma_{n}^{2}$. Note that the $\sigma_{n}^{2}$ is a noise variance from the data measurement. The covariance matrix consists of covariance values $k\left(x_{i},x_{j}\left|\theta \right.\right)$ defined by the covariance function or kernel. The covariance matrix $K\left(X,X\left|\theta \right.\right)$ for the training set is presented in the equation below with the parameter vector θ. The covariance matrix $K\left(X,X_{*}\left|\theta \right.\right)$ between the training and test sets is illustrated in the equation below as well.

(17) $$K\left(X,X\left|\theta \right.\right)=\left[\begin{array}{cccc} k\left(x_{1},x_{1}\left|\theta \right.\right) & k\left(x_{1},x_{2}\left|\theta \right.\right) & \cdots & k\left(x_{1},x_{L}\left|\theta \right.\right)\\ k\left(x_{2},x_{1}\left|\theta \right.\right) & k\left(x_{2},x_{2}\left|\theta \right.\right) & \cdots & k\left(x_{2},x_{L}\left|\theta \right.\right)\\ \vdots & \vdots & \ddots & \vdots \\ k\left(x_{L},x_{1}\left|\theta \right.\right) & k\left(x_{L},x_{2}\left|\theta \right.\right) & \cdots & k\left(x_{L},x_{L}\left|\theta \right.\right) \end{array}\right]\in {\mathbb{R}}^{L\times L}$$

(18) $$K\left(X,X_{*}\left|\theta \right.\right)=\left[\begin{array}{cccc} k\left(x_{1},x_{*1}\left|\theta \right.\right) & k\left(x_{1},x_{*2}\left|\theta \right.\right) & \cdots & k\left(x_{1},x_{*L_{*}}\left|\theta \right.\right)\\ k\left(x_{2},x_{*1}\left|\theta \right.\right) & k\left(x_{2},x_{*2}\left|\theta \right.\right) & \cdots & k\left(x_{2},x_{*L_{*}}\left|\theta \right.\right)\\ \vdots & \vdots & \ddots & \vdots \\ k\left(x_{L},x_{*1}\left|\theta \right.\right) & k\left(x_{L},x_{*2}\left|\theta \right.\right) & \cdots & k\left(x_{L},x_{*L_{*}}\left|\theta \right.\right) \end{array}\right]\in {\mathbb{R}}^{L\times L_{*}}$$

The kernel (or covariance function) denotes the similarity between two latent function variables $g\left(x_{i}\right)$ and $g\left(x_{j}\right)$ equivalently between data points $x_{i}$ and $x_{j}$ by a kernel trick [59]. The kernel develops the similarity model with parameters for the signal standard deviation $\sigma_{f}$, and characteristic length-scale l. Both parameters control the horizontal and vertical relationships between the points. Table 1 provides the conventional kernel functions with corresponding parameters.

After establishing the covariance matrix with the kernel function, the parameters $\beta ,\;\theta ,\mathrm{and}\;\sigma_{n}^{2}$ are necessary to be estimated from the given training dataset to complete Equation (14). The maximum posteriori estimate of the parameter occurs when the conditional probability distribution of the parameter is at its highest. With insufficient prior knowledge about the parameter, the highest corresponds to the maximizing point from the marginal log likelihood shown in the equation below.

(19) $$\log P(y|X,\beta ,\theta ,\sigma_{n}^{2})=-\frac{1}{2}{\left(y-h\beta \right)}^{T}{\left(K\left(X,X|\theta \right)+\sigma_{n}^{2}I_{L}\right)}^{-1}\left(y-h\beta \right)\;\begin{array}{c} -\frac{L}{2}\log\left(2\pi \right)-\frac{1}{2}\log\left|K\left(X,X|\theta \right)+\sigma_{n}^{2}I_{L}\right| \end{array}$$

The multivariate optimization algorithm [60,61,62] over Equation (19) executes the following equation to find the respecting parameters in a numerical way.

(20) $$\hat{\beta },\hat{\theta },{\hat{\sigma }}_{n}^{2}=\underset{\beta ,\theta ,\sigma_{n}^{2}}{\arg\;\max}\left(\log P(y|X,\beta ,\theta ,\sigma_{n}^{2})\right)$$

Once the kernel is selected, the parameters are estimated by the training process, and the predictions are performed by Equation (14) from the parameters. The prediction for a single point can be realized with the equation below.

(21) $$y_{*}=\bar{g}\left(x_{*}\right)=\beta +k\left(x_{*},X\right){\left(K\left(X,X\right)+\sigma_{n}^{2}I_{L}\right)}^{-1}\left(y-h\beta \right)$$

The GPR training and prediction require intensive computation as $O\left(N^{3}\right)$ due to the inversion of the covariance matrix. To release the complexity, numerous methods are possible, such as subsets of data point approximation [53,63], subsets of regressor approximation [53,64], etc. This paper applied the GPR without any approximation on training and prediction to remove any uncertainties caused by the information reduction. However, the optimized matrix inversion algorithms are used, such as LU decomposition [65] and Cholesky decomposition [66].

**Figure 4:**
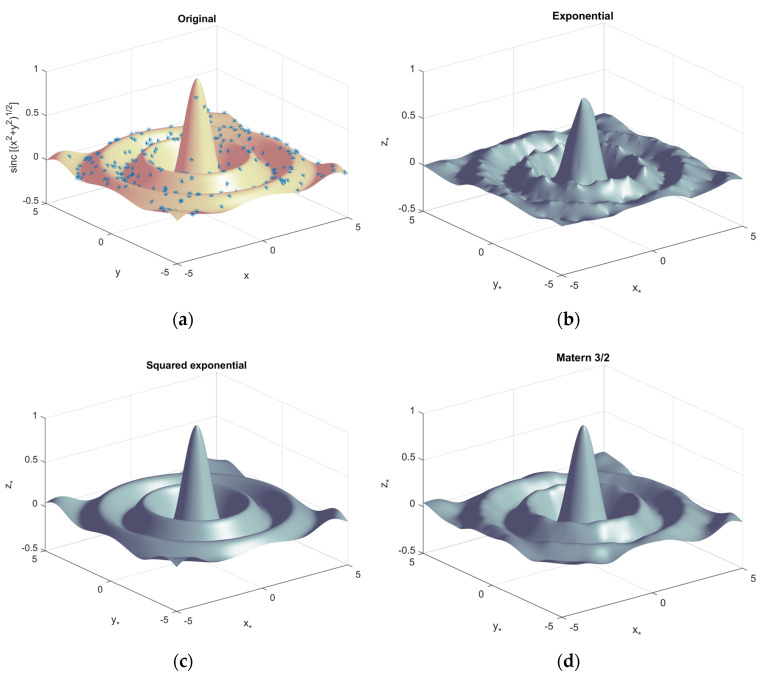
Gaussian process regression example by 3D sinc function. (Trained by 300 data points) (**a**) Original sinc function with one million data points. The * indicates training data points; (**b**) predictions by GPR with exponential kernel; (**c**) predictions by GPR with squared exponential kernel; (**d**) predictions by GPR with Matern 3/2 kernel.

The explained GPR is utilized for the predictions from the 3D sinc function in Figure 4. The original 3D sinc plot is depicted by the one million *x* and *y* grid points in Figure 4a. The GPR is trained by the randomly chosen 300 data points (* in Figure 4a), and the measurement is observed in the immaculate situation of the noise-free input data. The GPR with exponential, squared exponential, and Matern 3/2 kernels predicts the rest of the figure based on the estimated parameters in Figure 4b, Figure 4c, and Figure 4d, respectively. The GPR prediction performance suggests the significance of the kernel selection. If possible, prior knowledge of the data model leads to a better choice of the kernel function.

## 4. Simulations

In the previous section, the SCSSL algorithm is implemented by the HD, feature extraction, and GPR in consecutive order. This section realizes and performs the SCSSL algorithm over the computer-generated data. The performances are evaluated over the various parameters, such as kernels, orders, and ensemble lengths to determine the optimal choices for the actual experiments. Prior to the discuss of the SCSSL system simulation, the performance of the SCSSL system depends on the receiver configuration due to the derived time delay patterns from the receiver locations. The pattern diversity contributes to the prediction accuracy, and massive choices on the receiver configuration engage in the complex decision. This section initiates the simulation with the receiver configuration.

### 4.1. Receiver Locations

The HD algorithms compute the time delay between the receivers; hence, the receiver locations provide the performance fluctuation based on the temporal information quality. At least three receivers are required to resolve the angles for the π (180°) range. The higher numbers of receivers produce better performance in accuracy; however, the prediction range is still limited to π. The ambiguity originated from the HD algorithms, which introduced the range constraint. Observe that the time delays from the HDs are arranged for the positive values and cannot be distinguished between θ and θ+π in the given algorithm structure. Therefore, the receiver configuration comprises three receivers for π range observation.

**Figure 5:**
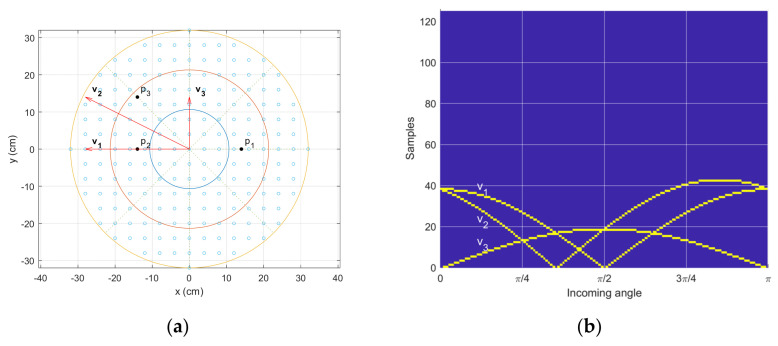
Receiver configuration and time delay distribution. (**a**) Receiver grid—black circles $\left(p_{1},\;p_{2},\;p_{3}\right)\;$indicate one example receiver location, and red arrows $\left(v_{1},\;v_{2},\;v_{3}\right)\;$ show the vectors between the receivers in the given configuration. (**b**) Time delay distribution for the given incoming angles. The yellow points provide the actual time delay (samples) created by the corresponding vectors $\left(v_{1},\;v_{2},\;v_{3}\right)$.

Figure 5 shows the arbitrary receiver configuration and corresponding time delay distribution. The receivers are placed on the particular grids (tiny blank circles in Figure 5a), which are located over the plane circle with a 32-cm radius. The distance between the adjacent grids is 4 cm in the horizontal and vertical directions for the total of 197 grids. Note that the discrete grids are applied to limit the number of combinations. The three receivers are located at $p_{1}$, $p_{2}$, and $p_{3}$ positions, and the resultant vectors are shown at $v_{1}\;\left(=p_{2}-p_{1}\right)$, $v_{2}\;\left(=p_{3}-p_{1}\right)$, and $v_{3}\;\left(=p_{3}-p_{2}\right)$ directions in Figure 5a. The receiver positions $p_{i}$ and vectors $v_{i}$ are represented by complex numbers in the Cartesian complex plane. Using the conventional angle notation, the arrivals from the east, north, and west directions correspond to the θ for 0, π/2, and π, respectively. For the individual vector $v_{i}$, the actual time delay $\tau_{i}$ in samples can be calculated by the following equation.

(22) $$\tau_{i}\left(\theta \right)=\frac{1}{cT_{s}}\mathrm{round}\left(\left|Re\left(v_{i}e^{j\theta }\right)\right|\right)$$

The θ is the arrival angle in radians, c is the sound speed (34,613 cm/s), $T_{s}$ is the sampling period (1/48,000 s), j is the imaginary number, and $Re\left(\right)$ is the operator for extracting the real part from the complex number. Note that the $\mathrm{round}\left(\right)$ in Equation (22) represents the round to the nearest integer function.

The equation below generates Figure 5b for the time delay distribution, where the time delay locations are identified with yellow points. The ***T*** matrix represents the time delay distribution, and the ***t*** vector indicates the time delays for individual angles.

(23) $$T_{nj}=\left[t_{1}\;t_{2}\ldots t_{A}\right]=\overset{A}{\underset{j=1}{\sum }}\overset{V}{\underset{i=1}{\sum }}\delta \left[n-\tau_{i}\left(\theta_{j}\right)\right]\;0\leq n\in \mathbb{Z}\leq n_{max}\;T\in {\mathbb{R}}^{n_{max}\times A}\;t\in {\mathbb{R}}^{n_{max}\times 1}$$

The $\delta \left[\;\right]$ is the Kronecker delta function with time n on a sample unit, *A* is the number of discrete angles, *V* is the number of time delay vectors, and $n_{max}$ is the maximum time delay on a sample for all configurations. Figure 5b casts the *n* for vertical and $\theta_{j}$ for horizontal axis.

The optimal receiver configuration can be obtained by employing the brute force method which systematically enumerates all feasible configurations for the least prediction error. The extensive computations are necessary for searching for the best receiver positions since each configuration realizes the entire SCSSL procedure for possible angles. By understanding the machine learning characteristics, the feature discrimination significantly mitigates the computational burden for evaluating the receiver configurations. The optimal receiver configuration should provide distinctive time delays on every assessing angle for information diversity. Observe that the GPR requires the unique feature for individual angles.

The diversity pattern can be analyzed by creating the similarity matrix ***S*** in the equation below. The multiplication in the equation below is the inner product between vectors.

(24) $$S_{ij}=\overset{A}{\underset{j=1}{\sum }}\overset{A}{\underset{i=1}{\sum }}t_{j}^{T}t_{i}\in {\mathbb{R}}^{A\times A}$$

**Figure 6:**
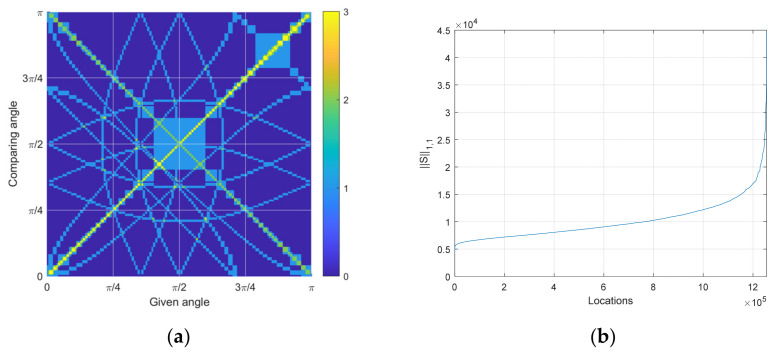
Similarity analysis. (**a**) The similarity matrix for the given receiver configuration shown is Figure 5a. The higher value indicates the higher similarity between the given and comparing angle; (**b**) The sorted distribution of similarity matrix $L_{1,1}$ norm for all possible combinations of three receiver configurations.

The ***t*** vector is from Equation (23), and the ***S*** matrix is illustrated in Figure 6a. According to the Cauchy–Schwarz inequality [67], the inner product with itself or the same pattern produces the highest values. Figure 6a demonstrates the highest values of the diagonal axis, which indicate the auto-inner products. The off-diagonal element should yield lower values than the diagonal elements; otherwise, the GPR delivers the confusion decision.

The ***S*** matrix is established for one receiver configuration. The $L_{1,1}$ norm of the ***S*** matrix shown below presents the overall similarity of the given configuration.

(25) $${∥S∥}_{1,1}=\overset{A}{\underset{i=1}{\sum }}\overset{A}{\underset{j=1}{\sum }}\left|s_{ij}\right|$$

The $L_{1,1}$ norm is the simple sum of all absolute elements. The lower value in ${∥S∥}_{1,1}$ denotes the higher diversity and equivalently, the lower similarity in time delay pattern since the off-diagonal elements are decreased overall. Note that the diagonal elements are predetermined by the auto-inner products as constants. Figure 6b illustrates the sorted $L_{1,1}$ norm of the ***S*** matrix for all receiver configurations. The combination of the three receiver positions resulting in 197 grid points is 1,254,890, which describes the *x* axis of Figure 6b.

**Figure 7:**
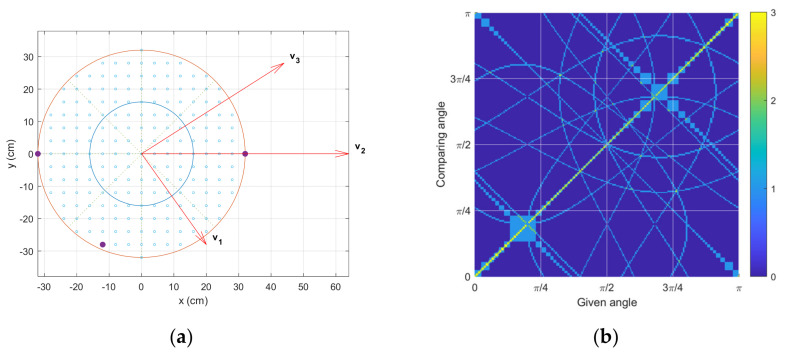
Optimal similarity outcome, which shows the lowest similarity matrix $L_{1,1}$ norm. (**a**) One of the best configurations for the three-receiver structure. (**b**) The corresponding similarity matrix.

The optimal receiver configuration is chosen from the least $L_{1,1}$ norm and is shown in Figure 7a. Multiple configurations provide identical minimum $L_{1,1}$ norm values due to the geometric symmetry of rotated structures. The receiver positions are located at the edge of the circle so that they can deliver the widest range of time delays in the pattern. Additionally, the asymmetricity is observed in the receiver positions to discriminate the east and west directions. Note that the symmetric receiver configuration provides the prediction of up to the π/2 range. The corresponding similarity matrix is depicted in Figure 7b. In contrast to Figure 6a, the optimal similarity matrix presents a substantially low distribution in Figure 7b. Fewer highest values are detected in the off-diagonal axis area in Figure 7b; therefore, improved accuracy is expected from the GPR prediction in the Figure 7a receiver configuration. The angle ambiguity is expected to be reduced for the wider receiver position with an increased receiver number.

**Figure 8:**
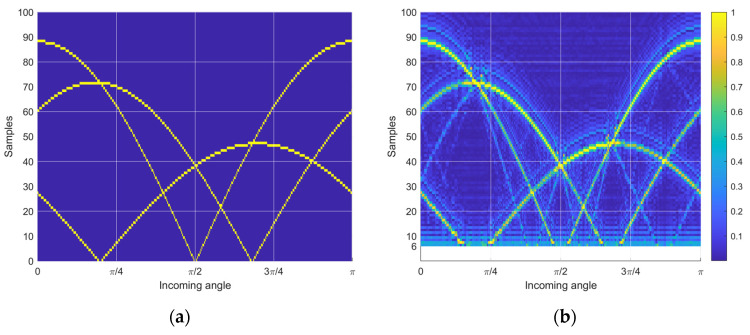
Time delay distribution from optimal similarity configuration. (**a**) Theoretical time delay distribution. (**b**) Estimated time delay distribution with nonparametric HD.

Figure 8a presents the time delay distribution for the optimal receiver configuration shown in Figure 7a. The dynamic range and the diversity pattern of the delay are further expanded and improved than in the example case, respectively. Corresponding to the similarity matrix in Figure 7b, minor pattern overlaps are observed in the distribution in Figure 8a. Figure 8b illustrates the estimated time delay distribution by using the nonparametric HD algorithm on the simulated data. The data is generated from the receiver’s perspective in the time domain with a 20 dB SNR. The estimated time delay precisely visualizes the theoretical time delay with trivial shadows caused by the DFT length limitation and noise interference [30]. Additionally, note that the minimum detectable time delay is determined by the window function in stage ⑤ of the HD algorithms shown in Figure 2. Figure 8b initiates the time delay axis (*y* axis) from six samples due to the five samples in the window length.

The optimal receiver configuration is justified by the similarity matrix and its corresponding norm. This process significantly reduces the computation of evaluating the receiver configuration. The previous paper [42] performed the 680-combination evaluation, the prediction error analysis, on 14 computers (intel i7-7700/32GB memory) with 25-h execution time. This paper assesses the 1,254,890 combinations of the single computer (inter i7-10700k/32GB memory) for less than half hour. The maximum diversity on the time delay pattern is provided with the receiver configuration and is interpreted properly with feature extraction and GPR.

### 4.2. Localization Performance

The SCSSL system sequentially executes HD, feature extraction, and GPR for prediction based on the provided receiver configuration. The performance of the SCSSL depends on the prediction accuracy and the independent variables are listed as ensemble length, parametric order, kernel functions, and incoming angles. The length of ensemble average controls the depth of average for enhancing the SNR in HD. The parametric order determines the pole and/or zero number to adjust peaks and valleys of the time delays to follow the distribution. The kernel functions define the relationship between the feature vectors to establish the prediction model. The incoming angle selects the training and evaluating angles for the performance analysis. This subsection analyzes the performance in terms of prediction accuracy with described independent parameters.

**Figure 9:**
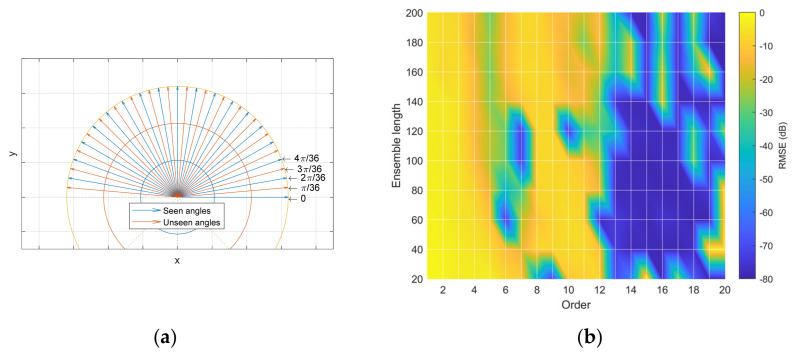
Incoming angles and RMSE distribution. (**a**) The angle distribution for learning is classified as seen angles (blue arrows) and the angles between the adjacent seen angles are organized as unseen angles (orange arrows). (**b**) One example of RMSE distribution in decibel scale for various orders and ensemble lengths.

The GPR produces the continuous output of the angle prediction because of the regression property; however, training and analyzing the system require discrete angles. The SCSSL system is trained by the seen angles from 0 to π−π/18 with π/18 resolution. Note that the opposite direction angles should be avoided to remove the confusion due to the π ambiguity in the HD algorithms. The angles between the adjacent seen angles are organized as the unseen angles to characterize the inference performance of the SCSSL system. The vocabulary ‘seen’ and ‘unseen’ is adopted to distinguish the included and excluded angles for learning, respectively, and both types of angles are shown in Figure 9a. The SCSSL system performance is numerated by root mean square error (RMSE), as shown below.

(26) $$RMSE\left(y\right)=\sqrt{\frac{1}{L}\overset{L}{\underset{i=1}{\sum }}{\left({\hat{y}}_{i}-y_{i}\right)}^{2}}\;y={\left\{y_{i}\right\}}_{i=1}^{L}\in {\mathbb{R}}^{L\times 1}$$

The $y_{i}$ is the real output (label) and ${\hat{y}}_{i}$ is the predicted output. For the given kernel function, the RMSE for the parametric HD method is illustrated by the colormap surface plot (shown in Figure 9b), with parametric order and an ensemble length parameter. The RMSE of the nonparametric HD is depicted by the conventional plot with ensemble length only.

The simulation parameters and corresponding values are arranged in Table 2. For one prediction, the SCSSL algorithm requires the ensemble length frames with overlapping between frames. Each frame consists of 1024 samples; however, the frame progresses only for the nonoverlapped samples, 256. The number of seen angles is equal to the number of unseen angle, and 0–π angles are called the angle set. The SCSSL algorithm is trained by the 111 angle sets with 1998 iterations. The validation process is performed by two angle sets of the training as 222 angle set and 3996 iterations. Note that one iteration is one angle training, and the training/validation are operated by the separated dataset.

**Figure 10:**
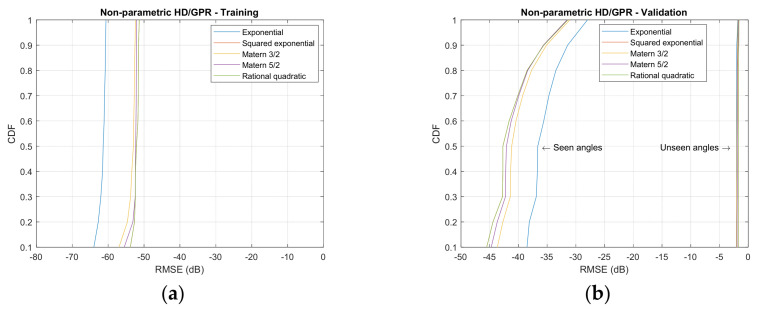

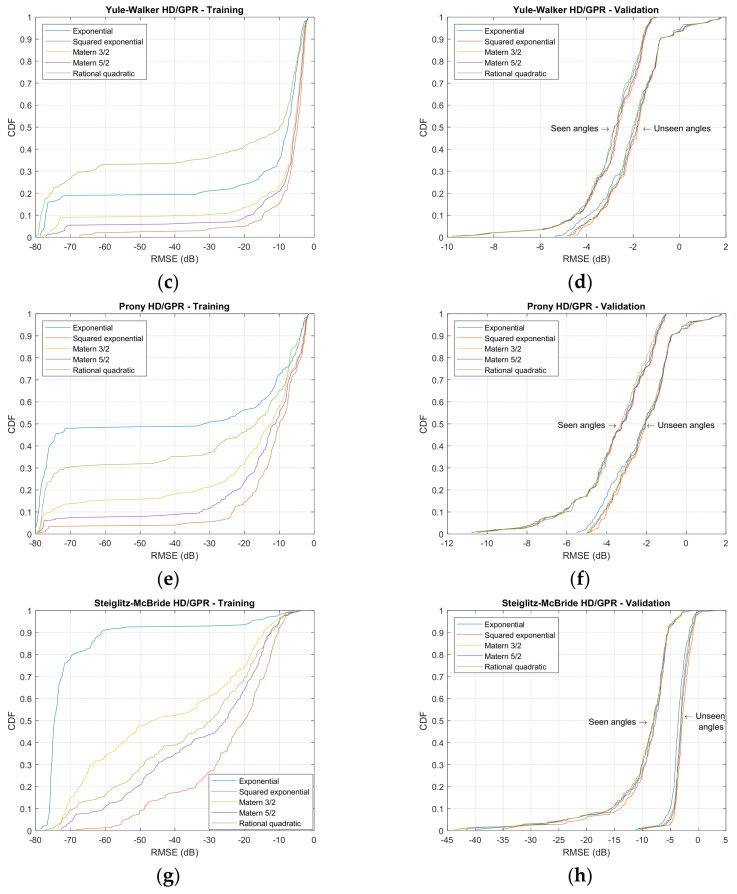
Empirical cumulative distribution function (CDF) distributions for simulated RMSE from GPR kernel functions. (Training: 1998 iterations; validation seen-angle: 3996 iterations; and validation unseen-angle: 3996 iterations.) (**a**) Nonparametric HD/GPR—Training. (**b**) Nonparametric HD/GPR—Validation; (**c**) Yule–Walker HD/GPR—Training; (**d**) Yule–Walker HD/GPR—Validation; (**e**) Prony HD/GPR—Training; (**f**) Prony HD/GPR—Validation; (**g**) Steiglitz–McBride HD/GPR—Training; (**h**) Steiglitz–McBride HD/GPR—Validation.

Figure 10 demonstrates the empirical cumulative distribution function (CDF) for simulated RMSE with kernel functions, HD algorithms, and angle classification on training and validation. The empirical CDF is constructed from the sorted RMSE in decibel and cumulated up to the individual RMSE for probability. The empirical CDF shown earlier, equivalently further left, indicates better performance in prediction because the reduced RMSEs are dominant. Eventually, all empirical CDF approaches the unit value due to the probability property. The speed represents the performance of the SCSSL algorithm. The left and right figures are divided into training and validation sets. Each row of the figure matrix is organized for the individual HD algorithms. The colored legend plots in the figure are designed to identify the kernel functions.

The nonparametric HD/GPR shows the consistent RMSE values for the training set in Figure 10a. The exponential kernel delivers the least RMSE distribution in the training set; however, the rational quadratic kernel is narrowly located on the left of the other kernels in validation and seen angles shown in Figure 10b. The unseen angles produce nearly identical distributions for all kernels. The Yule–Walker HD/GPR provides the distinguished empirical CDFs for individual kernels in training set in Figure 10c, and the rational quadratic kernel exhibits the optimal performance. According to the CDF slopes, the majority of the RMSEs are located at the edges of the performance graph. The validation in Figure 10d provides approximately duplicated performance for all kernels in seen and unseen angles. Unlike the training CDFs, the RMSEs are gathered at the center of the RMSE distribution by analyzing the slopes.

On Figure 10e, the Prony HD/GPR demonstrates the comparable performance to the Yule–Walker HD/GPR outcome for training. The steeper slopes are measured on low RMSE values for all kernels and the exponential kernel denotes the best performance in training. The validation in Figure 10f stays in Figure 10d for all kernels and seen/unseen angles; however, the Prony HD/GPR initiates the RMSE distribution slightly earlier. The Steiglitz–McBride HD/GPR delivers the prominent performance in training set, as shown in Figure 10g. The exponential kernel quickly increases the empirical CDF in low RMSE range for optimal performance and the other kernels consistently distributed the RMSE values based on the slope analysis. The validation in Figure 10h indicates the least RMSE distributions among the parametric HDs. The unseen angle performance is outstanding against all HD/GPR algorithms. Based on the investigation described above, the nonparametric HD/GPR, Yule–Walker HD/GPR, Prony HD/GPR, and Steiglitz–McBride HD/GPR chose the rational quadratic, rational quadratic, exponential, and exponential kernel, respectively.

**Figure 11:**
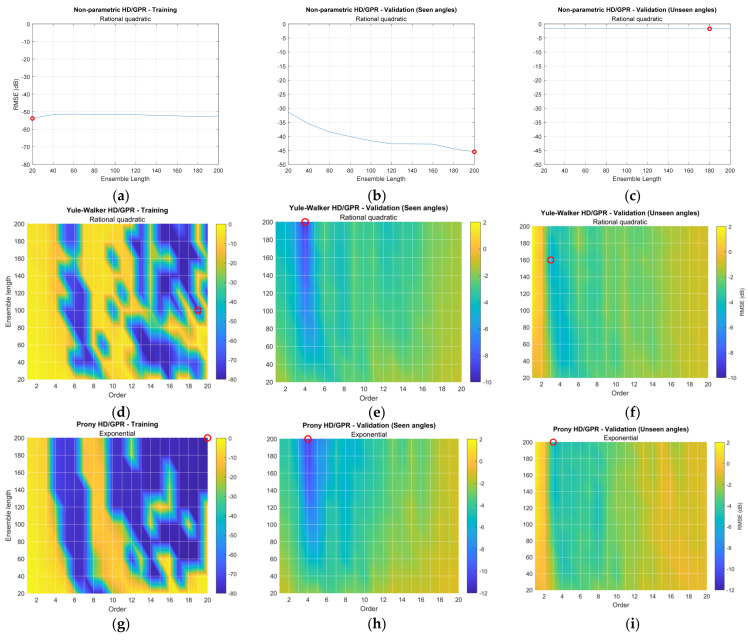

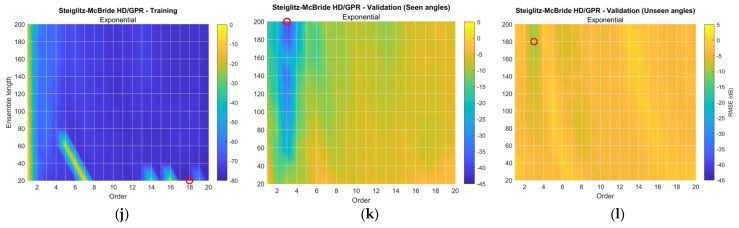
Simulated RMSE distribution for selected GPR kernel function. The red circle indicates the minimum RMSE location. (Training: 1998 iterations; validation seen-angle: 3996 iterations; and validation unseen-angle: 3996 iterations.) (**a**–**c**) Non-parametric HD/GPR—Training and validations; (**d**–**f**) Yule–Walker HD/GPR—Training and validations; (**g**–**i**) Prony HD/GPR—Training and validations; (**j**–**l**) Steiglitz–McBride HD/GPR—Training and validations.

Figure 11 provides the RMSE distribution for ensemble length and/or parametric order based on the selected kernel function from Figure 10. Each row of the figure matrix shows the individual HD/GPR, and each column specifies the training, validation for seen angles, and validation for unseen angles. The red circle in each figure corresponds to the lowest RMSE position to indicate the best parameters. The nonparametric HD/GPR is a function of the ensemble length only; therefore, the first row of Figure 11 is a conventional 2D plot. The parametric HD/GPR RMSEs are determined by the ensemble length as well as parametric order; hence, the figures except the first row are described by the 3D surface plot with color bars. Note that the surface plots are generated by the interpolation, and the grids crossed by the white lines represent the original simulated and noninterpolated positions. The depth of RMSE in decibels is equalized with Figure 10 simulation.

Figure 11a–c collect the RMSEs for non-parametric HD/GPR. Relatively consistent distributions were observed in all plots, and the validation for seen angles in Figure 11b provides the least RMSE on 200 ensemble lengths, which indicates the best parameter. Note that the best parameters should be evaluated during the validation process based on the hold-out dataset. Figure 11d–f arrange the RMSEs for Yule–Walker HD/GPR. The training RMSE distribution in Figure 11d illustrates the performance fluctuation in column-wise patterns. The high orders and around order 6 designate the low RMSE contributions. The validation RMSE for seen angles in Figure 11e shows the RMSE valley in order 4 and long ensemble length, which are recognized as the best parameters. Figure 11f for unseen angles includes the overall high RMSE distribution.

Figure 11g–i organizes the RMSEs for Prony HD/GPR. The overall distributions for training and validation resemble the Yule–Walker HD/GPR counterparts with a lower bias. Observe that the blue area in Figure 11g and the colorbar in Figure 11h,i correspond to the blue area in Figure 11d and the colorbar in Figure 11e,f, respectively. The best parameters for the Prony HD/GPR are order 4 and ensemble length 200 as well. Figure 11j–l consolidates the RMSEs for the Steiglitz–McBride HD/GPR. The training RMSE distribution in Figure 11j provides a low RMSE distribution for wide parameter combinations except for minor high glitches at certain spots. The validation RMSEs for seen and unseen angles in Figure 11k,l yield the RMSE valley at the order 3 column for higher ensemble length. The optimal parameters for Steiglitz–McBride HD/GPR are order 3 and ensemble length 200.

Table 3 summarizes the RMSE performance for the selected kernel function. The first column shows the SCSSL methods with kernel function in parentheses. The second column presents the best parameter values and corresponding RMSE in decibel for validation on seen angles. The third and fourth column collect the training and validation (unseen angles) RMSE on the determined best parameters, respectively. Note that the arrows in the column title indicate the source of the parameter. The RMSEs in squared brackets represent the best RMSE values which belong to the column simulation.

**Figure 12:**
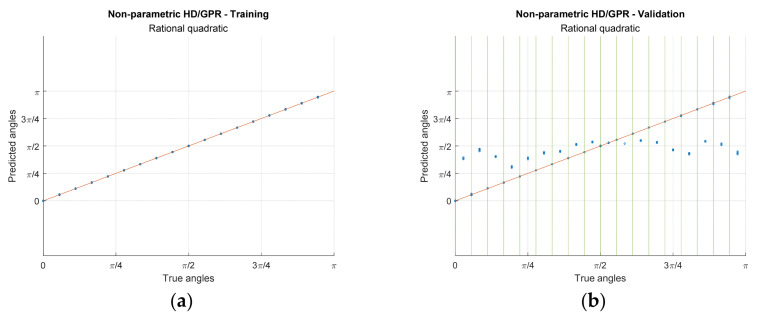

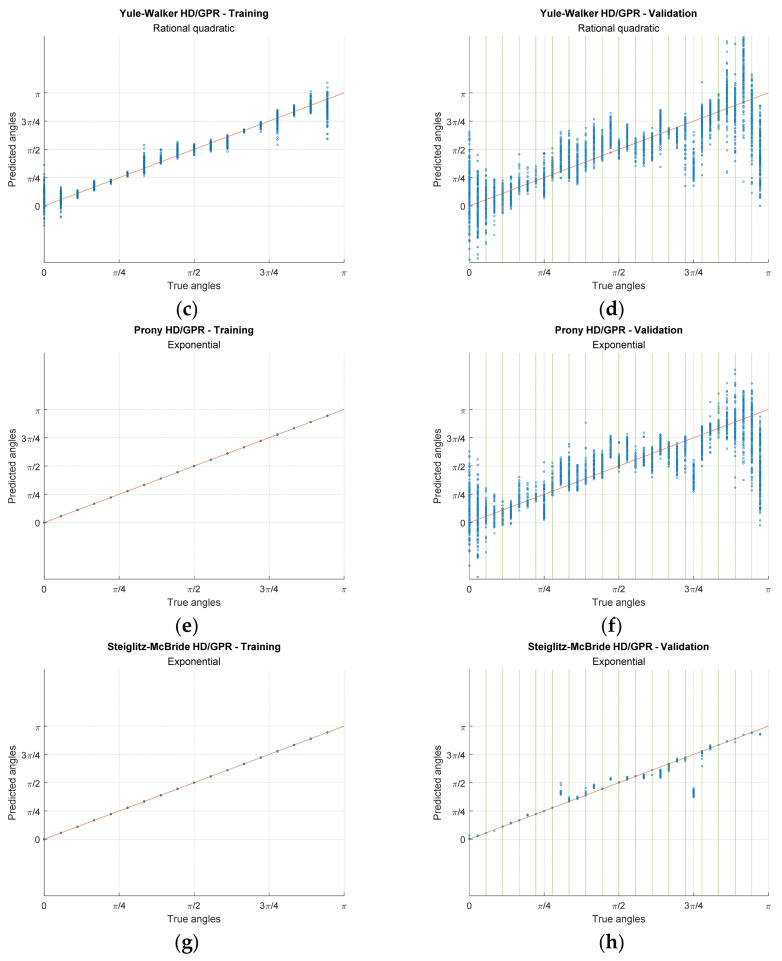
Actual predictions for training and validation from simulation. The iteration numbers are identical to Figure 9 and Figure 10 caption. The green vertical lines show the seen angles. (**a**,**b**) Non-parametric HD/GPR—Training and Validation; (**c**,**d**) Yule–Walker HD/GPR—Training and Validation; (**e**,**f**) Prony HD/GPR—Training and Validation; (**g**,**h**) Steiglitz–McBride HD/GPR—Training and Validation.

Figure 12 demonstrates the predictions from the HD/GPR algorithms for training and validation. The rows of the figure matrix specify the individual HD/GPR algorithms and the columns divide the training and validation. Figure 12 realizes the individual coordinate by x axis with real angles and y axis with predicted angles. The solid orange diagonal line in the figures indicates the perfect prediction. The 18 angular positions are isolated by π/18 radian distance with equiprobable possibility for all iterations in training. The 36 angles are evaluated on validation for seen and unseen angles with π/36 radian away from the adjacent angles. Note that the unseen angles (even sequence in validation angles) did not participate in the training process and seen angles are identified by the green vertical lines in validation plots. The single points in the plot are the cumulated predictions with very low prediction variance.

The non-parametric HD/GPR shows the coherent predictions for training and validation in Figure 12a,b, respectively. The calculated RMSEs for training and validation (seen angles) are −52.43 dB and −45.52 dB, individually, as shown in Table 3 with given optimal parameter. Therefore, the non-parametric HD/GPR predicts the seen angles with high accuracy and low variance. However, the unseen angle predictions in Figure 12b present the high bias and low variance in statistics. The predictions are located at approximately π/2 for all unseen angles with inferior RMSE performance −1.74 dB. The Yule–Walker HD/GPR provides the wide range of the predictions for training and validation in Figure 12c,d, respectively. The computed RMSEs for training and validation (seen angles) are −15.90 dB and −9.77 dB, individually, as shown in Table 3 with given best parameters. The Yule–Walker HD/GPR predicts the seen angles with low accuracy even for the training process because of the Yule–Walker estimator property [30]. The validation for seen angles denotes the wider prediction distribution than the training counterparts. The unseen angle predictions produce the RMSE as −3.99 dB which lends the better performance than the non-parametric HD/GPR since the prediction mean follows the true angles in Figure 12d.

The Prony HD/GPR derives the mixed prediction characteristics for training and validation in Figure 12e,f correspondingly. The evaluated RMSEs for training and validation (seen angles) are −71.69 dB and −10.79 dB, respectively, as shown in Table 3 with given best parameters. The Prony HD/GPR predicts the seen angles with high accuracy and low variance for the training in Figure 12e. The seen angle predictions in validation on Figure 12f generate the performance degradation with low accuracy and high variance which is comparable to the Yule–Walker HD/GPR equivalent. The unseen angle predictions provide the RMSE as −4.71 dB which offer the similar performance with the Yule–Walker HD/GPR because the prediction averages pursue the real angles as well in Figure 12f. The Steiglitz–McBride HD/GPR introduces the neat predictions for training and validation in Figure 12g,h, respectively. The calculated RMSEs for training and validation (seen angles) are −63.48 dB and −39.91 dB, individually, as shown in Table 3 with given best parameters. The Steiglitz–McBride HD/GPR estimates the seen angles with high accuracy and low variance for the training in Figure 12g. Additionally, the seen angle predictions in validation on Figure 12h deliver the consistent high precision arranged nearby the perfect prediction line. Notable property of the prediction is that the unseen angles are distinctly estimated for the true angles with low variation and −10.73 dB RMSE. The unseen angles approximately 11π/36 and 3π/4 demonstrate the biased estimation due to the duplication in the time delay pattern. The similarity matrix in Figure 7b validates the duplication by inspecting the off-diagonal high values nearby 11π/36 and 3π/4 angle. The Steiglitz–McBride HD/GPR provides the best performance for all angle situations with accurate inference.

This simulation section investigates the optimal receiver configuration and localization performance analysis based on the computer-generated dataset. The similarity matrix significantly reduces the computational complexity for finding the best configuration from numerous combinations. Based on the optimal receiver configuration, the localization performance is evaluated for kernel functions, ensemble length, parametric order, and directional angles. The Steiglitz–McBride HD/GPR with exponential kernel demonstrates the best performance for seen and unseen angles with various dataset. The hold-out dataset for unseen angles can be predicted with −10.73 dB RMSE. The minor prediction glitches are observed due to the receiver configuration limitation.

## 5. Results

The acoustic experiments are implemented and evaluated in an anechoic chamber, which is proved to show limited conformance with ISO 3745 [68] for the 1 kHz–16 kHz 1/3 octave band in a hemi-free-field mode and for the 250 Hz–16 kHz 1/3 octave band in a free-field mode [43]. The SCSSL methods are analyzed in the free-field mode which requires completely shielded surfaces for all directions with acoustic wedges. The acoustic experimentation demands three microphones with predetermined locations (shown in Figure 7a) and single speaker with far-field provision. The directional angle between receivers and speaker are guided by the line laser (GLL 3–80 P, Bosch, Gerlingen, Germany) located above the speaker. The far-field provision is preserved by maintaining a one-meter minimum distance for all possible signal propagations.

**Figure 13:**
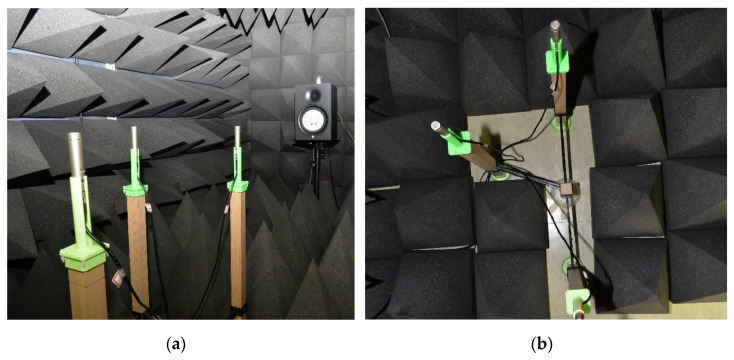
Acoustic experiment in the anechoic chamber: (**a**) three microphones and one speaker configuration with laser guidance; (**b**) top-view of the receiver structure and angular movement engager (the center pole).

The microphones are positioned at the receiver frame composed of lumbers with plastic supports. The structure retains the receiver shape and microphone height for circular movements. Note that the speaker and receiver level should be identical for unimpeded horizontal propagation. The circular movements for incoming angles involve the pair of saw-toothed wheels with engraved and intagliated shape for π/18 (10°) rotations. As photographed in Figure 13, the three receivers are placed in the radial pattern with concentric circles; hence, the circle center provides the angular movement center which is designed for the saw-toothed wheel pair. The general experiment arrangement is presented in Figure 13a and the receiver center is depicted in Figure 13b for the circular movement engager. The plastic accessories are realized by the 3D printer (Replicator 2, MakerBot, Brooklyn, NY, USA) from the polylactic acid (PLA) filament.

The analog mixer (MX-1020, MPA Tech, Seoul, Republic of Korea) combines the microphones (C-2, Behringer, Tortola, British Virgin Islands) for establishing the single channel signal. The computer-connected audio interface (Quad-Capture, Roland, Hamamatsu, Japan) quantizes the single-channel signal for SCSSL algorithms. The speaker (HS80M, Yamaha, Hamamatsu, Japan) is also connected to the audio interface to produce the wideband acoustic signal. The generation and reception of real-time audio are managed by the MATLAB system object with the audio stream input/output (ASIO) driver. The audio is recorded for 5 min in every appointed angles. The first and last one second of data are excluded to decrease the interruptions by transition conditions. Consequently, the experiment for individual angles utilizes approximately 5 min of recorded data. Each data frame is built by 1024 samples and the frame progresses to the further time by non-overlapped samples 256 for information consistency. The angular movement engager (shown in Figure 13b) is designed to rotate the receiver structure for every π/18 degree with the given 3D printer resource. Hence, the experiment angles are reduced by half for seen and unseen angle datasets. The experiment parameters are organized in Table 4.

**Figure 14:**
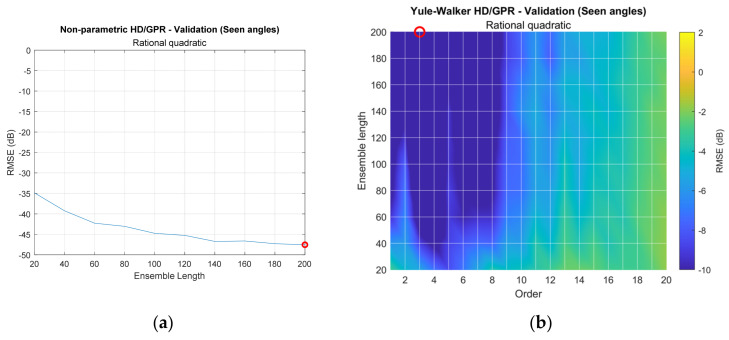

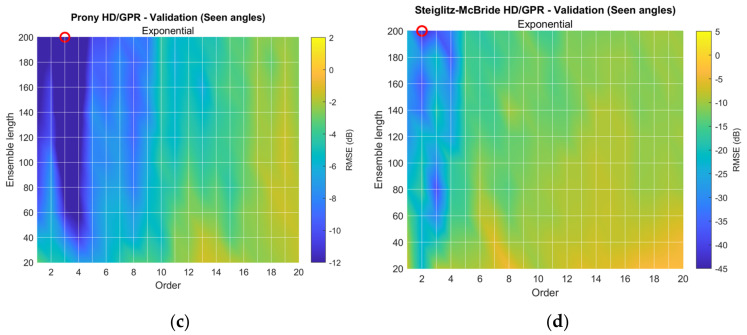
Experimented RMSE distribution for selected kernel functions. The red circle indicates the minimum RMSE location. (Training: 999 iterations; and validation seen-angle: 999 iterations.) (**a**) Nonparametric HD/GPR—Validation (seen angles); (**b**) Yule–Walker HD/GPR—Validation (seen angles); (**c**) Prony HD/GPR—Validation (seen angles); (**d**) Steiglitz–McBride HD/GPR –Validation (seen angles).

Figure 14 shows the experimented RMSE distributions with a selected kernel function which is derived from the simulation. The validation is performed with 9 seen angles for 999 training iterations and 999 validation iterations. The range of the RMSE is equalized to the previous RMSE distribution plots, such as in Figure 10 and Figure 11. The red circle on each figure corresponds to the lowest RMSE position to indicate the best parameters. The non-parametric HD/GPR demonstrates the gradually decreasing distribution from −35 dB to −48 dB. The improved SNR from the longer ensemble length contributes to the lower RMSE. The Yule–Walker HD/GPR indicates the enhanced RMSE on the area for longer ensemble length and low parametric order. The low RMSE area is wide and clustered. The Prony HD/GPR introduces the analogous RMSE distribution to the Yule–Walker HD/GPR counterpart. The low RMSE area is narrower than the lower parametric order. The Steiglitz–McBride HD/GPR delivers the low RMSE area on the small region with fragmented pattern. The orders 2, 3, and 4 with certain ensemble lengths produce very low RMSE values.

Table 5 organizes the best parameter values and corresponding RMSE from the validation process. The longer ensemble length achieves the lowest RMSE values for all HD/GPR algorithms. Observe that the optimal parametric orders are increased by one from the order chosen from the experimental RMSE distribution (seen angles) in Figure 14. The shifted orders are selected from the balance between the seen and unseen angle performance. Additionally, the parameter values are equal to the values from the simulation results shown in Table 3. The nonparametric HD/GPR produces the leading prediction accuracy for the seen angles according to the RMSE values. However, the unseen angle performance is significantly decreased, as expected from the prediction simulation shown in Figure 12b. The Steiglitz–McBride HD/GPR provides the lowest RMSE value for the unseen angles and the second RMSE performance for seen angles with least parametric order. The Yule–Walker and Prony HD/GPR methods show the overall performance deterioration for seen and unseen angles.

**Figure 15:**
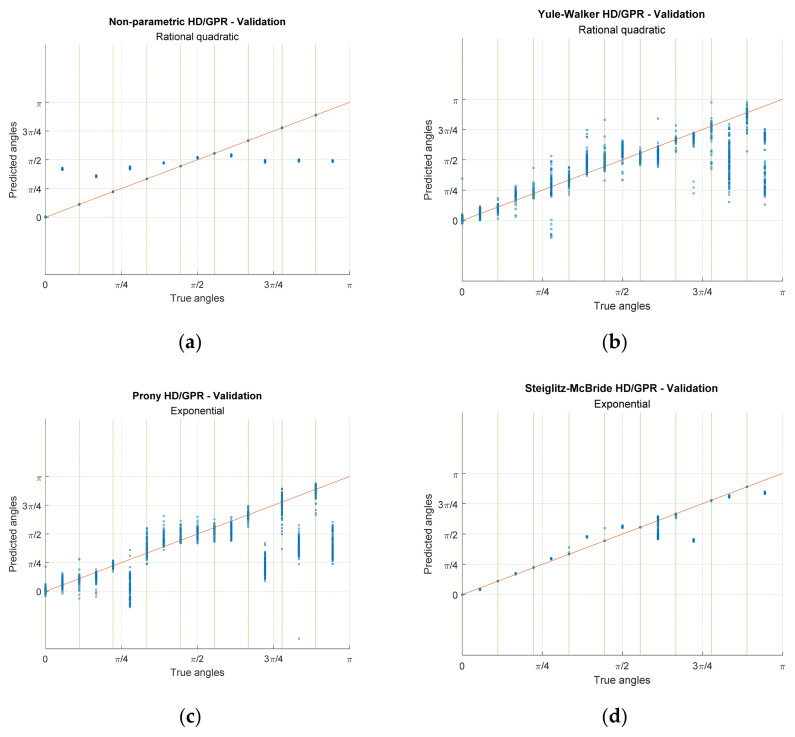
Actual predictions for validation from experiment. The iteration numbers are identical to Figure 13 caption. The green vertical lines show the seen angles. (**a**) Non-parametric HD/GPR—Validation; (**b**) Yule–Walker HD/GPR—Validation; (**c**) Prony HD/GPR—Validation; (**d**) Steiglitz–McBride HD/GPR—Validation.

Along with the previous simulations and experiments, this paper arrives at the final phase of investigating the actual prediction distribution based on the selected kernel function, ensemble length, and parametric order. Figure 15 shows the predictions from the individual HD/GPR algorithms for seen and unseen angles. The number of predictions is described in Table 4 as 999/999 iterations for seen/unseen angles. Note that the seen angles are indicated by the green vertical lines in the plots. In Figure 15a, the nonparametric HD/GPR represents the accurate predictions on seen angles with −47.54 dB RMSE; however, the predictions on unseen angles are consistent but biased with −1.95 dB RMSE. In Figure 15b, the Yule–Walker HD/GPR presents a wide variance on all predictions with −15.75 dB/−3.04 dB RMSE for seen/unseen angles. The means of the distribution seem to be following the perfect prediction line except for unseen angles around west end arrivals. In Figure 15c, the Prony HD/GPR provides a similar performance pattern to the Yule–Walker HD/GPR with −15.50 dB/−0.24 dB RMSE for seen/unseen angles. The performance glitches on the unseen angles are observed around west end and north-east arrivals. In Figure 15d, the Steiglitz–McBride HD/GPR demonstrates the overall best performance in terms of bias and variance with −37.56 dB/−9.11 dB RMSE for seen/unseen angles. The unseen angles around north-east (3π/4) arrivals provide the biased estimations due to the duplication in the time delay pattern shown in Figure 7b.

This results section performs the actual experiments with the optimal receiver configuration over the recorded audio from the anechoic chamber. With a wideband sound source, the three receivers are connected by the audio analog mixer and wired to the audio interface to collect data for individual angles. The HD/GPR algorithms show better performance with longer ensemble lengths and lower parametric orders, overall. The minimum RMSE value for each HD/GPR algorithm denotes the comparable performance outline to the simulation RMSE counterpart with minor discrepancy. The non-parametric HD/GPR and Steiglitz–McBride HD/GPR show the good performance on seen angles. The Steiglitz–McBride HD/GPR provides high prediction accuracy at unseen angles. The other HD/GPR algorithms generate marginal prediction performance for seen or unseen angles with high variance and/or high bias. Therefore, the Steiglitz–McBride homomorphic deconvolution, the pole angle method (feature extraction), and Gaussian process regress (exponential kernel) are combined as the SCSSL algorithm for optimal prediction on extensive incoming angles.

## 6. Conclusions

This paper provides the novel method to localize the angle of arrival based on the deconvolution with Gaussian process regression. The forward and backward homomorphic systems in cascade realize the deconvolution to extract the propagation function, which contains the time of flight between receivers. The nonparametric method is represented by the raw distribution of homomorphic deconvolution. The Yule–Walker, Prony, and Steiglitz–McBride from the spectral estimation technique are employed in the last stage of homomorphic deconvolution for model- and parameter-based propagation representation as a parametric method. The Gaussian process regression with a kernel (covariance) function calculates the mean and covariance of the updated function to predict the output based on the given training data. The Gaussian process regression predicts the angle from the given feature input once the learning process is completed. For feature extraction, the direct method and pole angle method are used for the nonparametric and parametric homomorphic deconvolutions, respectively. The optimal receiver configuration for a three-receiver structure is derived from the proposed similarity matrix analysis based on the time delay pattern diversity. The extensive simulations that present specific model order and longer ensemble length produce the least root mean square error. The Steiglitz–McBride model with an exponential kernel demonstrates the best performance for trained and untrained angles with various datasets. The experiments in the anechoic chamber present precise predictions with proper model order and ensemble length. The nonparametric method with a rational quadratic kernel shows good performance on trained angles. The Steiglitz–McBride model with an exponential kernel delivers the best predictions for trained and untrained angles based on the reduced model order.

This paper broadens the single-channel sound source localization system of the previous article to improve the comprehensive prediction performance by using Gaussian process regression with novel feature extraction. The foundation of the single channel sound source localization system was constructed by the time delay estimation using nonparametric and parametric homomorphic deconvolutions which was shown in prior independent article. The advantages of the system involve reduced system complexity because of the single analog receiver network with a single analog-to-digital converter for serving multiple receivers. The second benefit includes moderate modeling of machine learning algorithms to derive the estimation algorithm. Without using the complex propagation model, the training process of Gaussian process regression from the selected kernel function provides proper functioning for the sound source localization. Contrary to linear regression in the previous paper, the outcome of this paper proved that the Gaussian process regression with noble feature extraction realized the highest accuracy prediction for a wide range of the angles even for the untrained directions. Another enhancement of this system is introduced by the consistent algorithm complexity against receiver numbers. With fixed parameter values, the computational requirement is invariant to the receiver number. Observe that the conventional sound source localization systems show the same proportional complexity in terms of receiver numbers. The statistical analysis in simulation and experimentation demonstrates the feasibility of sound localization based on homomorphic deconvolution and Gaussian process regression. This paper contributes to increasing the possibility for the realization of a deployable and feasible sound source localization system by improving scalability and complexity. Currently, machine and deep learning techniques present aggressive progress for providing further accuracy in predictions and decisions with sparse or no datasets. The proposed system is expected to be improved by the use of advanced learning algorithms. Future work may aim to enhance the proposed system in complex situations, such as receiver failure and indirect propagation, etc. In addition, the future paper will explore the localization performance in the field using data collected by the mobile robot in a conventional environment. Due to the flexibility and expandability of deconvolution and machine learning, the use of the proposed method is not limited to sound source localization. From any sequential signals, the phase information on the multiple receivers can be extracted by the proposed algorithm. The future work will assist in finding additional utilizations other than sound source localization in succession.

## Figures and Tables

**Table 1:** Conventional kernel functions and corresponding parameters [53].

Kernel Name	$\mathrm{Kernel}\;\mathrm{Function}\;k\left(x_{i},x_{j}|\theta \right)$ with Parameter θ
Squared exponential	$k\left(x_{i},x_{j}|\theta \right)=\sigma_{f}^{2}\exp\left[-\frac{1}{2}\frac{{\left(x_{i}-x_{j}\right)}^{T}\left(x_{i}-x_{j}\right)}{l^{2}}\right]\;\theta =\left[\begin{array}{cc} l & \sigma_{f} \end{array}\right]$
Exponential	$k\left(x_{i},x_{j}|\theta \right)=\sigma_{f}^{2}\exp\left[-\frac{\sqrt{{\left(x_{i}-x_{j}\right)}^{T}\left(x_{i}-x_{j}\right)}}{l}\right]\;\theta =\left[\begin{array}{cc} l & \sigma_{f} \end{array}\right]$
Matern 3/2	$k\left(x_{i},x_{j}|\theta \right)=\sigma_{f}^{2}\left(1+\frac{\sqrt{3{\left(x_{i}-x_{j}\right)}^{T}\left(x_{i}-x_{j}\right)}}{l}\right)\exp\left[-\frac{\sqrt{3{\left(x_{i}-x_{j}\right)}^{T}\left(x_{i}-x_{j}\right)}}{l}\right]\;\theta =\left[\begin{array}{cc} l & \sigma_{f} \end{array}\right]$
Matern 5/2	$k\left(x_{i},x_{j}|\theta \right)=\sigma_{f}^{2}\left(1+\frac{\sqrt{5{\left(x_{i}-x_{j}\right)}^{T}\left(x_{i}-x_{j}\right)}}{l}+\frac{5{\left(x_{i}-x_{j}\right)}^{T}\left(x_{i}-x_{j}\right)}{3l^{2}}\right)\exp\left[-\frac{\sqrt{5{\left(x_{i}-x_{j}\right)}^{T}\left(x_{i}-x_{j}\right)}}{l}\right]$ $\theta =\left[\begin{array}{cc} l & \sigma_{f} \end{array}\right]$
Rational quadratic	$k\left(x_{i},x_{j}|\theta \right)=\sigma_{f}^{2}{\left(1+\frac{{\left(x_{i}-x_{j}\right)}^{T}\left(x_{i}-x_{j}\right)}{2\alpha l^{2}}\right)}^{-\alpha }\;\theta =\left[\begin{array}{ccc} l & \alpha & \sigma_{f} \end{array}\right]$

**Table 2:** Simulation parameters and values.

Parameter	Value	Parameter	Value
Sampling frequency	48,000 Hz	Angle range	0≤θ<π
Frame length	1024 samples	Angle resolution	π/18 rad (10°)
Overlap length	768 samples (2^10^–2^8^)	Seen angles	0, π/18, 2π/18, …
Sound speed	34,613 cm/s	Unseen angles	Seen angles + π/36
Number of receivers	3	# of seen angles	18 (=1 angle set)
SNR	20 dB	# of unseen angles	18 (=1 angle set)
HD window length	5 samples	Training iterations	1998 iter. (111 angle sets)
Max time delay (*n_max_*)	90 samples	Validation iter. (Seen angles)	3996 iter.(222 angle sets)
Audio source	Wideband signal ~12,000 Hz	Validation iter. (Unseen angles)	3996 iter.(222 angle sets)

**Table 3:** The RMSE values for the selected kernel function and parameters.

SCSSL Method (Kernel Function)	Validation (Seen Angles) Best Parameter Corresponding RMSE	Training RMSE ← @ Parameter ^1^ [Best]	Validation (Unseen Angles) RMSE ← @ Parameter ^1^ [Best]
Non-para. HD/GPR (Rat. quad.)	200 Len.	−52.43 dB [−53.83 dB]	−1.74 dB [−1.74 dB]
	−45.52 dB		
YW HD/GPR (Rat. quad.)	4 Ord. and 200 Len.	−15.90 dB [−79.78 dB]	−3.99 dB [−4.88 dB]
	−9.77 dB		
Prony HD/GPR (Exponential)	4 Ord. and 200 Len.	−71.69 dB [−80.06 dB]	−4.71 dB [−5.49 dB]
	−10.79 dB		
SM HD/GPR (Exponential)	3 Ord. and 200 Len.	−63.48 dB [−75.77 dB]	−10.73 dB [−11.32 dB]
	−39.91 dB		

^1^ Best parametric order and ensemble length from the validation (seen angles).

**Table 4:** Experiment parameters and values.

Parameter	Value	Parameter	Value
Sampling frequency	48,000 Hz	Angle range	0≤θ<π
Frame length	1024 samples	Angle resolution	π/9 rad (20°)
Overlap length	768 samples (2^10^–2^8^)	Seen angles	0, π/9, 2π/9, …
Sound speed	34,613 cm/sec	Unseen angles	Seen angles + π/18
Number of receivers	3	# of seen angles	9 (=1 angle set)
SNR	Not applicable	# of unseen angles	9 (=1 angle set)
HD window length	5 samples	Training iterations	999 iter. (111 angle sets)
Max time delay (*n_max_*)	90 samples	Validation iter. (Seen angles)	999 iter. (111 angle sets)
Audio source	Wideband signal ~12,000 Hz	Validation iter. (Unseen angles)	999 iter. (111 angle sets)

**Table 5:** Minimum RMSE values for HD/GPR experiments and corresponding parameters.

SSL Method (Kernel Function)	Parameter	Validation (Seen Angles) RMSE	Validation (Unseen Angles) RMSE
Non-para. HD/GPR (Rat. quad.)	200 Len.	−47.54 dB	−1.95 dB
YW HD/GPR (Rat. quad.)	4 Ord. and 200 Len.	−15.75 dB	−3.04 dB
Prony HD/GPR (Exponential)	4 Ord. and 200 Len.	−15.50 dB	−0.24 dB
SM HD/GPR (Exponential)	3 Ord. and 200 Len.	−37.56 dB	−9.11 dB

## Data Availability

The acoustic data collected in the anechoic chamber for various angles are available upon request.

## Abbreviations

The following abbreviations are used in this manuscript (listed in order of appearance in the text).

SSL	Sound source localization
AoA	Angle of arrival
3D	Three-dimensional
ADC	Analog-to-digital converter
SCSSL	Single-channel sound source localization
HD	Homomorphic deconvolution
ToF	Time of flight
GPR	Gaussian process regression
DFT	Discrete Fourier transform
FFT	Fast Fourier transform
SNR	Signal-to-noise ratio
AR	Autoregressive
ARMA	Autoregressive moving average
RMSE	Root mean square error
CDF	Cumulative distribution function
PLA	Polylactic acid
ASIO	Audio stream input/output
